# MAC Performance Analysis for Reliable Power-Line Communication Networks with ARQ Scheme

**DOI:** 10.3390/s21010196

**Published:** 2020-12-30

**Authors:** Sheng Hao, Huyin Zhang

**Affiliations:** 1 School of Computer Science, Central China Normal University, Wuhan 430079, China; 2 School of Computer Science, Wuhan University; Wuhan 430079, China; 2008301500139@whu.edu.cn

**Keywords:** reliable power-line communication networks, medium access control, IEEE 1901 protocol, ARQ scheme, channel type, noise feature, performance analysis

## Abstract

Power-line communication (PLC) networks have been increasingly used for constructing industrial IoT (internet of things) and home networking systems due to their low-cost installation and broad coverage feature. To guarantee the transmission reliability, ARQ (automatic repeat request) scheme is introduced into the link layer of reliable PLC networks, which allows the retransmission of a data frame several times so that it has a higher probability to be correctly received. However, current studies of performance analysis for PLC MAC (medium access control) protocol (i.e., IEEE 1901) do not take into account of the impact of ARQ scheme. To resolve this problem, we propose an analytical model to investigate the MAC performance of IEEE 1901 protocol for reliable PLC networks with ARQ scheme. In the modeling process, we first establish a PLC channel model to reflect the impacts of PLC channel types (containing Rayleigh fading and Log-normal fading), additive non-Gaussian noise feature and ARQ scheme on data transmission at link layer. Next, we employ Renewal theory and Queueing dynamics to capture the transmission attempt behavior of executing IEEE 1901 protocol in the unsaturated environment with finite transit buffer size. On the basis of combining these two models, we derive the closed-form expressions of 1901 MAC metrics considering the influence of the ARQ scheme. Furthermore, we prove that the proposed analytical model has the convergence property. Finally, we evaluate the MAC performance of 1901 protocol for reliable PLC networks with ARQ scheme and verify the proposed analytical model.

## 1. Introduction

The past decade has witnessed a rapid development of IoT (Internet of things), which provides convenience for consumers, manufacturers, and public welfare [1,2,3,4]. Due to the limitation of battery life and wireless propagation, wireless communication (WLC) solution cannot accommodate all IoT requirements. For this purpose, industry and academia have developed power-line communication (PLC) networks as another promising technology for constructing IoT systems [4,5,6,7,8]. The reliable PLC network generally introduces some network coding schemes [9,10,11,12,13,14,15] into its link layer to ensure communication reliability, and the most typical one is the AqRQ (automatic repeat request) scheme. It allows a station to retransmit a data packet several times at link layer to enhance the received probability. Because of the introduction of the ARQ scheme, the performance of MAC (medium access control) protocol for reliable PLC networks i.e., IEEE 1901 [16,17,18,19,20], would be necessarily affected. In addition, the PLC channel fading type and additive non-Gaussian noise feature also influence 1901’s performance.

### 1.1. Drawbacks and Motivation

In recent years, some meaningful studies have been proposed to analyze the MAC performance of 1901 protocol [21,22,23,24,25,26,27,28,29]. However, all these works merely concentrate on formally describing the MAC layer’s CSMA/CA (Carrier Sense Multiple Access/Collision Avoidance) mechanism of 1901, and neglect how the PLC link layer’s ARQ scheme affects 1901’s MAC performance. They also assume the PLC network has an ideal channel, i.e., the data transmission failure would not be caused by the channel, which of course cannot comprehensively reflect the impacts of detailed PLC channel fading type and additive non-Gaussian noise feature [13]. In other words, there is a wide gap between these models and practical MAC performance behavior of reliable PLC networks. Moreover, these works fail to investigate whether their established models have the convergence property. Therefore, it is still a great challenge for us to propose an analytical model with insightful understanding, which can accurately evaluate the MAC performance of IEEE 1901 under the influence of ARQ scheme, channel fading type and additive non-Gaussian noise feature, and prove this model has the convergence property.

At the engineering application level, the data rate of current PLC networks can reach up to 1.5 Gbps [16,18], thus providing a high-speed communication medium. Up to now, PLC networks have enjoyed widespread success in commercial area. More than 200 million PLC-based devices are used around the world, which fully demonstrates that the PLC technology is a robust and cost-optimized mechanism. Many manufacturers (Panasonic, Huawei, Siemens etc.) have started to develop and design embedded PLC-based devices and smart chips (following IEEE 1901 protocol) [18]. Existing PLC-based IoT applications have related to diverse domains including Smart Grid/Home/Meter, Underground power transmission, Intelligent Mining, in-vehicle communication, photovoltaic system, hybrid intelligence communication and Industry 4.0, which greatly boost the development of our society and enhance the efficiency of our daily affairs. Hence, making thorough analysis of IEEE 1901 protocol for reliable PLC networks with ARQ scheme has practical value for guiding IoT system construction.

### 1.2. Contributions

The core contributions of the paper are as follows:

- We propose a MAC performance analysis model of IEEE 1901 protocol for reliable PLC networks with ARQ scheme. To accurately reflect the impacts of ARQ scheme, detailed PLC channel fading types (containing Rayleigh fading and Log-normal fading) [30,31,32,33,34] and additive non-Gaussian noise feature (following Bernoulli-Gaussian distribution [13]), we first construct a PLC channel model. It can be used to derive the probability that one transmission attempt process of PLC link fails. Then we use Renewal theory and Queueing dynamics to capture the transmission attempt behavior of executing IEEE 1901 protocol in unsaturated environment with finite transit buffer size. On the base of combining these two models, we derive the closed-form expressions of system throughput, mean MAC service time, buffer blocking probability, successful transmission probability etc. for reliable PLC networks with ARQ scheme.
- We demonstrate the proposed analytical model has the convergence property, and find out the theoretically optimal value of successful transmission probability.
- We conduct extensive simulation experiments to verify the proposed analytical model for reliable PLC networks with ARQ scheme.

### 1.3. Paper Outline

The rest of our paper is organized as follows: Section 2 provides the overview of IEEE 1901 and ARQ scheme. Section 3 reviews Related works. The system model is presented in Section 4. We verify the analytical model via simulations in Section 5. We conclude our paper in Section 6.

## 2. Overview of IEEE 1901 and ARQ Scheme

### 2.1. IEEE 1901 Protocol

1901 protocol employs a backoff counter and a deferral counter. The backoff counter process (BCP) of 1901 is basically same as that of 802.11, namely the station stars from the backoff stage 1 and the selected backoff counter is set to the value in $\left[1,...,W_{1}\right]$. When a slot is sensed idle, the backoff counter is decreased by 1; otherwise, it is frozen [23]. As the backoff counter decreases to 1, the station attempts to transmit the packet. When the station suffers a collision, it jumps to backoff stage 2 and repeats the same process. If the station is already at the last backoff stage *m* and suffers a collision, it would re-enter this backoff stage. Furthermore, 1901 has only four backoff stages (i.e., m=4), and it defines four priority classes (CA0−CA3) [16], where CA0/CA1 (or CA2/CA3) constitutes one priority type (the value of $W_{k}$ [16] is shown in Table 1).

The deferral counter process (DCP) of 1901 runs as following. When the station enters backoff stage *k*, the initial deferral counter is set to $d_{k}+1$ (the value of $d_{k}$[16] is shown in Table 1). When sensing the medium busy, the station decreases $d_{k}+1$ by 1 (executing the BCP at the same time). If the deferral counter reaches to 1 and the medium is sensed busy, it would enter backoff stage k+1 (if already at backoff stage *m*, it re-enters this stage) [23].

### 2.2. ARQ Scheme

**Figure 1:**
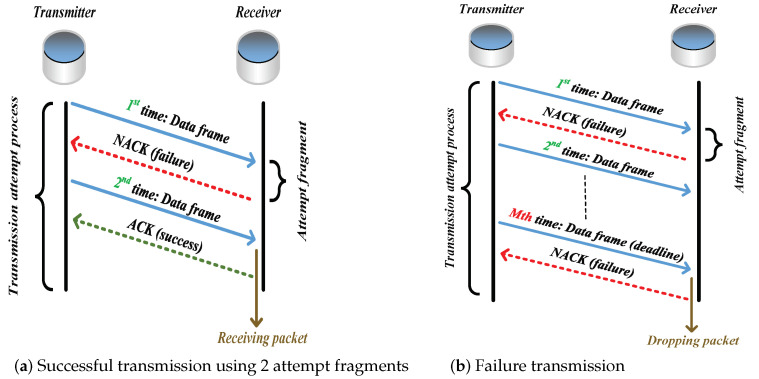
Transmission attempt process using ARQ scheme.

As a station occupies the channel (through executing 1901’s CSMA/CA mechanism) and attempts to transmit packets [25,26,27,28], it begins to trigger the ARQ scheme. For the receiver, it uses the packet received in the successful attempt fragment only, and discards all the previously erroneous copies of this packet (caused by unsuccessful attempt fragments). If the signal-to-noise ratio (SNR) of the packet received in an attempt fragment is less than the required minimum SNR (i.e., threshold SNR), the attempt fragment is unsuccessful, i.e., the packet is received in outage. One transmission attempt process contains no more than *M* attempt fragments. After each attempt fragment, the station receives an acknowledgment (ACK) frame if the packet is successfully received; otherwise, a negative ACK (NACK) frame. The packet would be dropped if it cannot be successfully delivered by the deadline (i.e., *M*th attempt fragment). Figure 1 shows a successful transmission attempt process using 2 fragments and a failure transmission attempt process.

## 3. Related Work

Until now, some significant studies have paid attention to analyze the MAC performance of IEEE 1901 protocol. In [21], Jung proposed a semi-Markov-based analytical model to characterize the CSMA/CA mechanism of 1901 protocol in saturated conditions. After that, Vlachou et al. put forward a series of analytical models for IEEE 1901 protocol using Renewal theory and strong law of large number (SLLN) [22,23,24,25,26], which can be used in the saturated environment. In [22,23], the authors constructed the basic analytical model and discussed the impact of deferral counter; In [24], they examined the performance tradeoff between delay and throughput; In [25], they further extended their previous work [23,24] to optimize 1901 MAC performance; Moreover, they analyzed the performance and stability of 1901 protocol under the coupling condition [26]. Similar to the work [23], Cano et al. mentioned a theoretical model for IEEE 1901 protocol in saturated conditions using renewal process theorem [27]. In [28], Hao et al. provided a unified theoretical framework for 1901 protocol, which can be used in both homogeneous and heterogeneous environment. Furthermore, they built a theoretical model for IEEE 1901 protocol in the multi-hop environment [29]. As previously said, the above-mentioned works do not consider the influence of PLC link layer’s ARQ scheme, detailed PLC channel fading type and additive non-Gaussian noise feature on the MAC performance of 1901. In addition, they fail to prove whether the proposed models have the convergence property. Table 2 summarizes the comparison between previous works and our current study for 1901 protocol analysis, containing mathematics tool and consider factors (*Y* denotes the considered factors, *N* the neglected factors).

## 4. System Model

**Figure 2:**
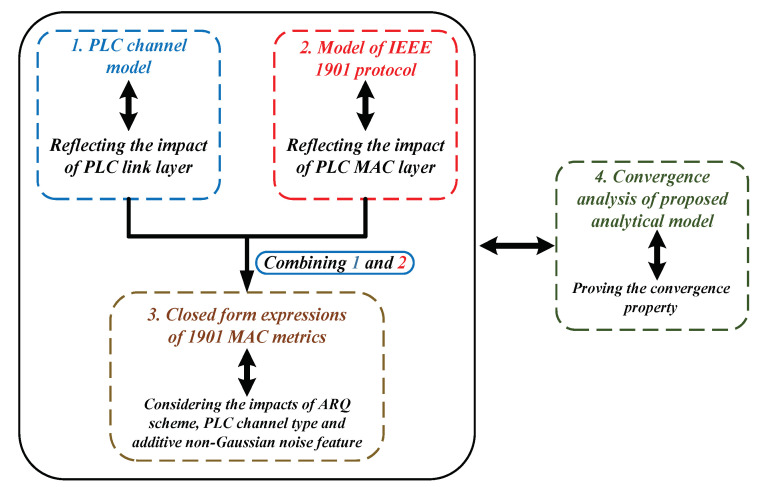
Modeling steps of the proposed analytical model.

In this part, we construct the analytical model of IEEE 1901 protocol for reliable PLC networks with ARQ scheme (the notation of essential results derived by the theoretical model is given in Table 3). For analytical tractability, we assume the reliable PLC network follows a single-hop, star-shaped topology, where all stations transmit their packets to the access point (AP). The modeling process is divided into four steps: (1) Establishing the PLC channel model to reflect the impacts of channel fading types, additive non-Gaussian noise feature and ARQ scheme on data transmission at link layer; (2) Providing a Renewal theory and Queueing dynamics-based model to depict the transmission attempt behavior of executing 1901 protocol in unsaturated environment with finite transit buffer; (3) Deriving the closed-form expressions of 1901’s MAC metrics for reliable PLC networks with ARQ scheme; (4) Proving the convergence property of our proposed analytical model (the structure of modeling steps is shown by Figure 2).

### 4.1. PLC Channel Model

#### 4.1.1. Rayleigh Fading Channel

In general, a reliable PLC network employs a Rayleigh fading channel or a Log-normal fading channel [30,31]. We first consider the situation that the PLC channel follows Rayleigh fading distribution, thus the PDF (probability density function) of PLC channel gain $f_{R}\left(h_{R}\right)$ can be written as:

(1) $$f_{R}\left(h_{R}\right)=\frac{h_{R}}{\sigma_{R}^{2}}\cdot exp\left(-\frac{h_{R}^{2}}{2\sigma_{R}^{2}}\right)$$

where $h_{R}$ represents the channel gain with Rayleigh distribution, $\sigma_{R}$ the scale parameter of Rayleigh distribution.

Since PLC noise contains background noise and potentially impulsive noise, following the Bernoulli-Gaussian distribution (i.e., the additive non-Gaussian noise feature) [13,34] the PDF of PLC noise distribution $f_{z}\left(z\right)$ can be represented as

(2) $$f_{z}\left(z\right)=\left(1-P_{I}\right)\cdot N\left(0,\sigma_{g}^{2}\right)+P_{I}\cdot N\left(0,\sigma_{b}^{2}\right)$$

where $\sigma_{g}^{2}$ and $\sigma_{b}^{2}$ are the variances of the background noise and impulsive noise component, respectively; $P_{I}$ signifies the occurrence probability of the impulsive noise component. $\sigma_{g}^{2}$ and $\sigma_{b}^{2}$ have a ration relation, i.e., $\sigma_{b}^{2}=\left(1+K\right)\sigma_{g}^{2}$. K is the ratio of the impulsive noise power.

Let Ptr be the transmission power, hence the SNR ξ of reliable PLC networks can be expressed as:

(3) $$\xi =\left\{\begin{array}{c} \frac{Ptr\cdot |h_{R}^{2}|}{\sigma_{g}^{2}}\;w.p.\;1-P_{I}\\ \frac{Ptr\cdot |h_{R}^{2}|}{\sigma_{b}^{2}}\;w.p.\;P_{I} \end{array}\right.$$

Directly applying general communication model [13,30,31], Ptr can be expanded as $Ptr=\Omega_{1}+\Omega_{2}\cdot X^{-\alpha }$, where $\Omega_{1}$ and $\Omega_{2}$ are two constants, *X* the transmission distance, and α the loss exponent.

The PDF of SNR for reliable PLC networks using Rayleigh fading channel $f_{\xi }^{R}\left(\xi \right)$ can be accordingly given by

(4) $$\begin{array}{cc} \; & f_{\xi }^{R}\left(\xi \right)=\frac{\left(1-P_{I}\right)}{2\sigma_{R}^{2}\cdot \frac{Ptr}{\sigma_{g}^{2}}}\cdot exp\left(-\frac{\xi }{2\sigma_{R}^{2}\cdot \frac{Ptr}{\sigma_{g}^{2}}}\right)+\frac{P_{I}}{2\sigma_{R}^{2}\cdot \frac{Ptr}{\sigma_{b}^{2}}}\cdot exp\left(-\frac{\xi }{2\sigma_{R}^{2}\cdot \frac{Ptr}{\sigma_{b}^{2}}}\right) \end{array}$$

Based on the above formal representation, we can write the CDF (Cumulative Distribution Function) of SNR using Rayleigh fading channel $F_{\xi }^{R}\left(\xi \right)$ as

(5) $$\begin{array}{cc} \; & F_{\xi }^{R}\left(\xi \right)=1-\left(1-P_{I}\right)\cdot exp\left(-\frac{\xi }{2\sigma_{R}^{2}\cdot \frac{Ptr}{\sigma_{g}^{2}}}\right)-P_{I}\cdot exp\left(-\frac{\xi }{2\sigma_{R}^{2}\cdot \frac{Ptr}{\sigma_{b}^{2}}}\right) \end{array}$$

#### 4.1.2. Log-Normal Fading Channel

Secondly, we consider the PLC channel following Log-normal fading distribution, and the PDF of PLC channel gain $f_{L}\left(h_{L}\right)$ can be expressed as:

(6) $$f_{L}\left(h_{L}\right)=\frac{1}{\sqrt{2\pi \sigma_{L}^{2}}\cdot h_{L}}\cdot exp\left(-\frac{{\left(\ln\left(h_{L}\right)-\mu_{L}\right)}^{2}}{2\sigma_{L}^{2}}\right);h_{L}>0$$

where $h_{L}$ denotes the channel gain with Log-normal distribution, $\sigma_{L}$ and $\mu_{L}$ the scale parameters of Log-normal distribution.

On the basis, the PDF of SNR ξ for reliable PLC networks using Log-normal fading channel $f_{\xi }^{L}\left(\xi \right)$ can be represented as (considering the additive non-Gaussian noise feature)

(7) $$\begin{array}{cc} \; & f_{\xi }^{L}\left(\xi \right)=\left(1-P_{I}\right)\cdot \frac{1}{\sqrt{2\pi \sigma_{L^{\prime }}^{2}}\cdot \xi }\cdot exp\left(-\frac{{\left(\ln\left(\frac{\sigma_{g}^{2}\xi }{Ptr}\right)-\mu_{L^{\prime }}\right)}^{2}}{2\sigma_{L^{\prime }}^{2}}\right)+P_{I}\cdot \frac{1}{\sqrt{2\pi \sigma_{L^{\prime }}^{2}}\cdot \xi }\cdot exp\left(-\frac{{\left(\ln\left(\frac{\sigma_{b}^{2}\xi }{Ptr}\right)-\mu_{L^{\prime }}\right)}^{2}}{2\sigma_{L^{\prime }}^{2}}\right) \end{array}$$

where $\sigma_{L^{\prime }}^{2}=4\sigma_{L}^{2}$, $\mu_{L^{\prime }}=2\mu_{L}$.

Then, the CDF of SNR for reliable PLC networks using Log-normal fading channel $F_{\xi }^{L}\left(\xi \right)$ can be derived as

(8) $$\begin{array}{cc} \; & F_{\xi }^{L}\left(\xi \right)=\frac{\left(1-P_{I}\right)}{2}\cdot erfc\left(\frac{\mu_{L^{\prime }}-\ln\left(\frac{\sigma_{g}^{2}\xi }{Ptr}\right)}{\sqrt{2\sigma_{L^{\prime }}^{2}}}\right)+\frac{P_{I}}{2}\cdot erfc\left(\frac{\mu_{L^{\prime }}-\ln\left(\frac{\sigma_{b}^{2}\xi }{Ptr}\right)}{\sqrt{2\sigma_{L^{\prime }}^{2}}}\right) \end{array}$$

where erfc(.) is the complimentary error function [31].

Through the above derivation, the probability that the transmission of PLC link fails, i.e., the outage probability $P_{o}$ can be expressed as

(9) $$P_{o}=Prb\left\{\xi <\zeta \right\}=\left\{\begin{array}{c} F_{\xi }^{R}\left(\zeta \right)\\ F_{\xi }^{L}\left(\zeta \right) \end{array}\right.$$

where ζ denotes the required minimum SNR for successful decoding, i.e., threshold SNR.

#### 4.1.3. ARQ Scheme

Since the reliable PLC network introduces ARQ scheme at link layer, one transmission attempt process is divided into several attempt fragments (no more than *M* fragments). Thus, for a reliable PLC network using ARQ scheme at link layer, the probability that one transmission attempt process of PLC link successes and needs *x* attempt fragments $P_{s}\left(x\right)$ can be written as

(10) $$P_{s}\left(x\right)=P_{o}^{x-1}\cdot \left(1-P_{o}\right);x\in \left[1,M\right]$$

Accordingly, for a reliable PLC network using ARQ scheme at link layer, the probability that one transmission attempt process of PLC link fails $P_{f}$ can be given by

(11) $$P_{f}=1-\sum_{x=1}^{M}P_{s}\left(x\right)=P_{o}^{M}$$

### 4.2. The Model of IEEE 1901 Protocol

In this part, we construct the model of IEEE 1901 protocol to depict the transmission attempt behavior at MAC layer.

The model is constructed under the following assumptions:

- There are *N* stations transmitting their data packets over the PLC channel. The HOL (head of line) packet transmission attempt process of each station follows decoupling assumption [23].
- The transit buffer of a station is finite (denoted by *K*). Packets would be blocked if the transit buffer is already fully loaded (denoted by buffer blocking probability $P_{b}$).
- Packets arrive at the station based on Poisson process with an average arrival rate λ. The time of generating a packet copy is short enough and can be neglected. In addition, we assume the generating packet copy is immediately forwarded without using the transit buffer space (i.e., a packet and its copies only need 1 slot of the buffer).

#### 4.2.1. The Transmission Attempt Probability of 1901 Protocol in Unsaturated Environment

First, we apply the results of the theoretical model established by [23] (suited for the saturated PLC environment), the transmission attempt probability of saturated environment-based 1901 protocol $\tau_{s}\left(\rho \right)$ can be expressed using Renewal theory, i.e.,

(12) $$\left\{\begin{array}{c} \tau_{s}\left(\rho \right)=E\left[R\right]\left(\rho \right)/E\left[X\right]\left(\rho \right);\;\\ E\left[R\right]\left(\rho \right)=\frac{1}{\left(1-\rho \right)};\\ E\left[X\right]\left(\rho \right)=\frac{1}{\sum_{i=1}^{m-1}b_{i}\prod_{j=1}^{i-1}\chi_{j}+\prod_{j=1}^{m-1}\chi_{j}\frac{b_{m}}{\left(1-\chi_{m}\right)}} \end{array}\right.$$

where ρ is the conditional collision probability of saturated environment. E[R](ρ) and E[X](ρ) denote the expected number of attempts by executing one complete procedure of IEEE 1901 protocol and the expected number of time slots spent in executing one complete procedure of 1901 (note that E[R](.) and E[X](.) are the functions of conditional collision probability). $b_{i}$ represents the expected number of time slots spent by a station at backoff stage *i*. $\chi_{i}$ signifies the probability that a station at backoff stage *i* must jump to the next stage (caused by unsuccessful attempt or DCP of 1901). They can be respectively represented as:

(13) Xri=∑t=di+1rrtρt(1−ρ)r−t;bi=1Wi∑r=di+1Wi[r(1−Xri)]+1Wi∑r=di+1Wi∑h=di+1kh[Xhi−Xh−1i]+∑r=1dirWi;χi=1−[∑r=1di1Wi+∑r=di+1Wi(1−Xri)]+ρ·[∑r=1di1Wi+∑r=di+1Wi(1−Xri)]

In Formula (13), *r* is defined as the initial selected backoff counter at stage *i*, *h* the time slots spent due to DCP of 1901. $X_{h}^{i}$ the probability of triggering DCP of 1901 and spending no more than *h* time slots at stage *i*.

**Remark** **1.** *The detailed derivation process of $\tau_{s}\left(\rho \right)$, E[R](ρ) and E[X](ρ) has been shown in [23], here we do not explain anymore. In addition, we can also adopt our previous work [28] (Markov chain model) to derive $\tau_{s}\left(\rho \right)$.*

Since we consider an unsaturated environment with finite transit buffer, the impact of buffer state should be taken into consideration. Let $P_{n}$ be the steady state probability that a station buffer is non-empty and *p* be the conditional collision probability in unsaturated environment, thus the transmission attempt probability $\tau_{u}\left(p\right)$ of 1901 in unsaturated environment can be given by

(14) $$\tau_{u}\left(p\right)=P_{n}\cdot \tau_{s}\left(p\right)=P_{n}\cdot \frac{E[R](p)}{E[X](p)}$$

The probability $p_{tr}$ that at least one station tries to a transmission in the considered slot time, and the probability $p_{idle}$ that the PLC channel is idle can be expressed respectively as:

(15) $$p_{tr}=1-{\left(1-\tau_{u}\left(p\right)\right)}^{N}$$

(16) $$p_{idle}=1-p_{tr}={\left(1-\tau_{u}\left(p\right)\right)}^{N}$$

If one of the other N−1 remaining stations attempts to transmit the HOL packet at the same slot time, a collision can be triggered (PLC networks follow the half-duplex communication mode). Hence, we construct the **fixed-point equation** to represent the relationship between $\tau_{u}\left(p\right)$ and *p*, i.e.,

(17) $$\begin{array}{c} p=1-{\left[1-\tau_{u}\left(p\right)\right]}^{N-1}=1-{\left[1-P_{n}\cdot \tau_{s}\left(p\right)\right]}^{N-1}=1-{\left[1-P_{n}\cdot \frac{E[R](p)}{E[X](p)}\right]}^{N-1} \end{array}$$

**Remark** **2.** *Although we have given the expression of $\tau_{u}\left(p\right)$, $P_{n}$ is still unknown, and it relies on the size of transit buffer K and detailed packet arrival mode. Thus, in the next part, we employ Queueing dynamics to express $P_{n}$.*

#### 4.2.2. The Derivation of $P_{n}$ Based on Queueing Dynamics

**Figure 3:**
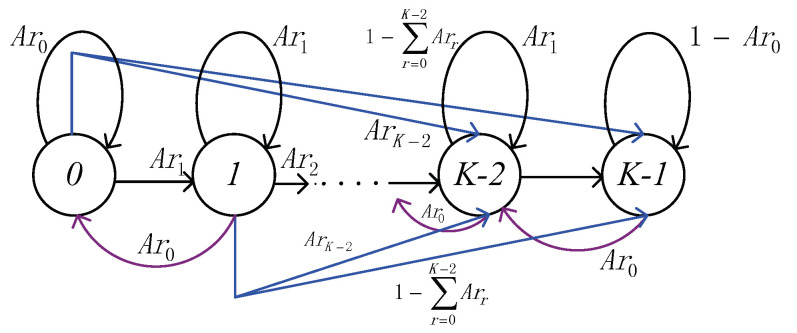
The relationship between $\pi_{a}$ and $Ar_{a}$.

To express $P_{n}$, we first investigate the Queueing behavior of the transit buffer. Let $p_{a}$ be the probability of *a* packets in the transit buffer, $\pi_{a}$ be the probability that there are *a* packets in the transit buffer after a packet departure, and $Ar_{a}$ be the probability that *a* packets arrive the station during the MAC service time $T_{mac}$[29] (the relationship between $\pi_{a}$ and $Ar_{a}$ is shown in Figure 3). Then we denote the state transition matrix Ar as

(18) $$\begin{array}{cc} \; & \mathrm{Ar}=\\ \; & \left[\begin{array}{cccccc} Ar_{0} & Ar_{1} & Ar_{2} & \cdots & Ar_{K-2} & 1-\sum_{a=0}^{K-2}Ar_{a}\\ Ar_{0} & Ar_{1} & Ar_{2} & \cdots & Ar_{K-2} & 1-\sum_{a=0}^{K-2}Ar_{a}\\ 0 & Ar_{0} & Ar_{1} & \cdots & Ar_{K-3} & 1-\sum_{a=0}^{K-3}Ar_{a}\\ \vdots & \vdots & \vdots & \ddots & \vdots & \vdots \\ 0 & 0 & 0 & \cdots & Ar_{0} & 1-Ar_{0} \end{array}\right]. \end{array}$$

Moreover, let $\pi =\left[\begin{array}{cccc} \pi_{0} & \pi_{1} & \cdots & \pi_{K-1} \end{array}\right]$ ($\sum_{a=0}^{K-1}\pi_{a}=1$), we can express the relationship between π and Ar as following

(19) π·Ar=π

Let E[slot] be the expected slot time, according to the Poisson arrival feature, $Ar_{a}$ used in Formulas (18) and (19) can be written as (Prb{.} denotes the probability that the case happens)

(20) $$\begin{array}{cc} \; & Ar_{a}=\sum_{j=0}^{\infty }\frac{exp\left(-\lambda jE\left[slot\right]\right)\cdot \;{\left(-\lambda jE\left[slot\right]\right)}^{a}}{a!}\cdot Prb\left\{T_{mac}=j\cdot E\left[slot\right]\right\} \end{array}$$

We further consider how to express the mean MAC service time $E[T_{mac}]$. Applying **Z transforms** (i.e., Generating function method) [35,36], the generating function of initial selected backoff counter *j* as $f_{1_{k}}\left(Z\right)$ and $f_{2_{k}}\left(Z\right)$ can be respectively defined as:

(21) $$f_{1_{k}}\left(Z\right)=\sum_{j=1}^{d_{k}}\frac{\left(Z^{jE[slot]}\right)}{W_{k}};k\in \left[1,m\right]$$

(22) $$f_{2_{k}}\left(Z\right)=\sum_{j=d_{k}+1}^{W_{k}}\frac{\left(Z^{jE[slot]}\right)}{W_{k}};k\in \left[1,m\right]$$

Combining with the parameter $\chi_{i}$ (defined in Formulas (12) and (13)), the probability generating function (PGF) of MAC service time F(Z) for reliable PLC network can be written as (for k=1, $\prod_{t=1}^{k-1}\chi_{t}=1$)

(23) $$\begin{array}{cc} \; & F\left(Z\right)=\sum_{k=1}^{m-1}\left[f_{1_{k}}\left(Z\right)\cdot \prod_{t=1}^{k-1}\chi_{t}+\left(1-X_{j}^{t}\right)\cdot f_{2_{k}}\left(Z\right)\cdot \prod_{t=1}^{k-1}\chi_{t}\right]+\\ \; & \prod_{t=1}^{m-1}\chi_{t}\left[f_{1_{m}}\left(Z\right)\cdot \frac{1}{1-\chi_{m}}+\left(1-X_{j}^{t}\right)\cdot f_{2_{m}}\left(Z\right)\cdot \frac{1}{1-\chi_{m}}\right] \end{array}$$

Formula (23) can be expanded as

(24) $$F\left(Z\right)=\sum_{j=0}^{\infty }Prb\left\{T_{mac}=jE\left[slot\right]\right\}Z^{jE[slot]}$$

Based on the knowledge of **Z transforms** [35,36], we can get the arbitrary moment of $T_{mac}$ by differentiating F(Z). Thus, the mean MAC service time $E[T_{mac}]$ can be expressed as

(25) $$E\left[T_{mac}\right]=\frac{dF(Z)}{dZ}|Z=1$$

**Remark** **3.** *Recalling Formulas (21) and (22) the generating function of initial selected backoff counter j is divided into two parts $f_{1_{k}}$ and $f_{2_{k}}$. The reason is that when $j<d_{k}+1$, a station cannot trigger the DCP of 1901, while $j\geq d_{k}+1$, a station would trigger the DCP of 1901 if it senses the medium busy $d_{k}+1$ times.*

$Ar_{a}$ in Formula (20) can be expanded as

(26) $$Ar_{a}=\frac{\lambda^{a}}{{\left(-1\right)}^{a}\cdot a!}\cdot \frac{\partial^{a}F\left(exp\left(-\lambda \right)\right)}{\partial \lambda^{a}}$$

We define the **traffic intensity** as Υ, and it can be given by

(27) $$\Upsilon =\lambda \cdot E[T_{mac}]$$

Using the conclusion of M/G/1/K queue model [37,38], the steady state probability $p_{a}$ of *a* packets in the transit buffer can be expressed as

(28) $$\left\{\begin{array}{c} p_{a}=\frac{\pi_{a}}{\pi_{0}+\Upsilon }\;a\in \left[0,K-1\right]\\ p_{K}=1-\frac{1}{\pi_{0}+\Upsilon } \end{array}\right.$$

Through the above derivation, the steady state probability that a station buffer is non-empty $P_{n}$ can be finally represented as

(29) $$P_{n}=\sum_{a=1}^{K}p_{a}=1-p_{0}=1-\frac{\pi_{0}}{\pi_{0}+\Upsilon }$$

In other words, the steady state probability that a station buffer is empty equals to $p_{0}$.

**Remark** **4.** *Reviewing the above derivation process, $P_{n}$ is expressed as a function of $Ar_{a}$ and $E[T_{mac}]$; however, $Ar_{a}$ and $E[T_{mac}]$ are functions of E[slot] (i.e., $P_{n}$ cannot simplified as a unary function of p), whose expression has not been obtained. It is governed not only by IEEE 1901 protocol but also by the ARQ scheme at link layer. Therefore, in the next subsection, we would combine the PLC channel model and IEEE 1901 model to derive the closed-form expressions of E[slot] and other significant MAC metrics for reliable PLC networks with ARQ scheme.*

### 4.3. The MAC Metrics of 1901 Considering ARQ Scheme

Due to the link layer’s ARQ scheme, the station can successfully transmit its HOL packet if and only if it holds the following two conditions ($c_{1}$ and $c_{2}$):

$c_{1}$: The station occupies the PLC channel through executing the 1901 protocol, and attempts to transmit its HOL packet;

$c_{2}$: The transmit attempt process of PLC link successes.

Therefore, the successful transmission probability $P_{suc}$ of reliable PLC networks can be expressed as

(30) $$\begin{array}{c} P_{suc}=N\cdot \underset{I}{\underbrace{\tau_{u}\left(p\right)\left(1-p\right)}}\underset{\mathrm{II}}{\underbrace{\cdot \sum_{x=1}^{M}P_{s}\left(x\right)}}=N\tau_{u}\left(p\right)\cdot \left(1-p\right)\cdot \left(1-P_{f}\right) \end{array}$$

In Formula (30), part “I” denotes the probability of meeting condition $c_{1}$, part “II” the probability of meeting condition $c_{2}$. *N* means the successful transmission happens among one of the *N* stations. Furthermore, it is easy to find that $P_{suc}$ has the theoretically optimal value (see the analysis in Appendix A).

Similarly, the probability that the packet is finally dropped in one transmission attempt process $p_{drop}$ can be expressed as

(31) $$p_{drop}=N\tau_{u}\left(p\right)\left(1-p\right)\cdot P_{o}^{M}=N\tau_{u}\left(p\right)\left(1-p\right)\cdot P_{f}$$

Reviewing the ARQ scheme, one transmission attempt process can be divided into *x* fragments (x∈[1,M]), if the packet attempt fails in a fragment, the receiver replies a NACK frame, else an ACK frame. Thus, based on the specification of 1901 protocol [16,18], the time duration that one successful transmission needs *x* fragments $T_{s}\left(x\right)$ is given by

(32) $$\begin{array}{cc} \; & T_{s}\left(x\right)=2PRS+\left(x-1\right)\cdot \left(D+RIFS+NACK\right)+D+RIFS+ACK+CIFS;\;x\in \left[1,M\right] \end{array}$$

The corresponding probability that a station successfully transmits its HOL packet and uses *x* fragments $p_{suc}\left(x\right)$ can be expressed as (note that $P_{suc}=\sum_{x=1}^{M}p_{suc}\left(x\right)$)

(33) $$p_{suc}\left(x\right)=N\tau_{u}\left(p\right)\cdot \left(1-p\right)\cdot P_{s}\left(x\right);\;x\in \left[1,M\right]$$

**Figure 4:**
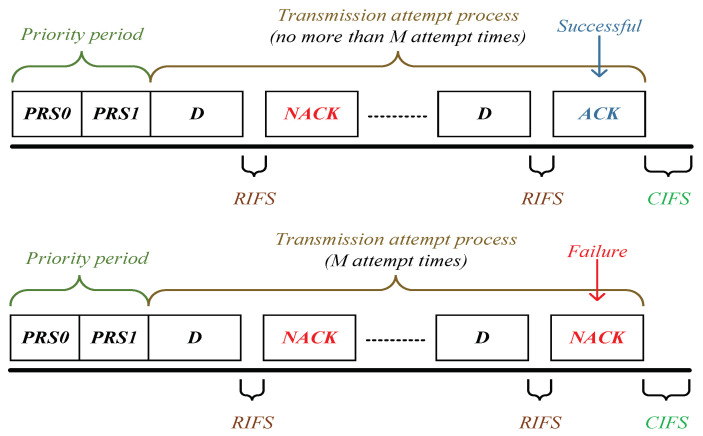
1901’s time sequence for reliable PLC networks with ARQ scheme.

Similarly, the time duration of “dropping packet” $T_{drop}$ for a station is derived as (the time sequence is shown in Figure 4)

(34) $$T_{drop}=2PRS+M\cdot \left(D+RIFS+NACK\right)+CIFS$$

The time duration due to the collision $T_{c}$ is defined as

(35) $$T_{c}=EIFS$$

In Formula (32), (34) and (35), PRS, RIFS, CIFS, ACK, NACK, *D* and EIFS denote the priority tone slot, response inter-frame space, contention inter-frame space, acknowledgment frame, negative acknowledgment frame, frame duration of data packet and extended inter-frame space, respectively.

Since a generic slot duration depends on whether a slot is idle or interrupted by a successful transmission or a failure transmission (“dropping packet”) or a collision, we can define slot as

(36) $$slot=\left\{\begin{array}{c} \sigma \;w.p.\;p_{idle}\\ T_{s}\left(x\right)\;w.p.\;p_{suc}\left(x\right);x\in \left[1,M\right]\\ T_{drop}\;w.p.\;p_{drop}\\ T_{c}\;w.p.\;\left(p_{tr}-P_{suc}-p_{drop}\right) \end{array}\right.$$

where σ is the idle slot.

Thus, the expected slot time E[slot] can be written as (a unary function of *p*)

(37) $$\begin{array}{c} E\left[slot\right]=\sigma p_{idle}+\sum_{x=1}^{M}T_{s}\left(x\right)p_{suc}\left(x\right)+T_{dp}p_{drop}+T_{c}\left(p_{tr}-P_{suc}-p_{drop}\right) \end{array}$$

The system throughput of reliable PLC networks with ARQ scheme *S* is accordingly given by

(38) $$\begin{array}{c} S=\frac{P_{suc}\cdot L}{E[slot]} \end{array}$$

where *L* represents the size of data packet.

In addition, the buffer blocking probability $P_{b}$ is represented as

(39) $$P_{b}=p_{K}=1-\frac{1}{\pi_{0}+\Upsilon }$$

Through the above derivation, E[slot] is finally written as a unary function of conditional collision probability *p* (since $Ar_{a}$ and $E[T_{mac}]$ are represented as the polynomials of *p*). Consequently, $P_{n}$ can be expanded as a polynomial of *p*, i.e., the right side of Formula (17) is a polynomial of *p*. Now we can assert that *p* and other significant MAC metrics are **solvable** in form.

### 4.4. Convergence Analysis of the Proposed Model

In this section, we analyze whether the proposed model has the convergence property. Since the overall analytical model needs to be solved through the **fixed-point equation** (Formula (17)), we should judge the convergence of the fixed point *p* to guarantee the proposed model’s solvability.

Reviewing Formula (17), we rewrite it as

(40) $$p=\Gamma (\tau_{u}\left(p\right))$$

Constructing a relaxed fixed-point sequence iteration equation [39,40] for the fixed point *p*, i.e.,

(41) $$p_{\alpha +1}=\left(1-\beta \right)\Gamma \left(\tau_{u}\left(p_{\alpha }\right)\right)+\beta p_{\alpha };\beta \in \left(0,1\right)$$

where α represents the iteration times.

Based on the convergence theory [39,40], the sequence $p_{\alpha }$ converges to a general fixed point *p*, if the first-order derivative of $\Gamma (\tau_{u}\left(p\right))$ is bounded and $\Gamma \left(\tau_{u}\left(p_{\alpha }\right)\right)\leq p_{\alpha }$.

Now we prove the above two constrained conditions hold.

**Theorem** **1.** *The first-order-derivative of $\Gamma (\tau_{u}\left(p\right))$ is bounded.*

**Lemma** **1.** *The first-order-derivative of $P_{n}$ is bounded.*

**Proof.**  To degrade the difficulty of analysis, we denote $p_{0}$ by an approximate expression [38] instead of using Formula (28) (directly using Formula (28) would greatly enhance the difficulty of mathematical derivation), i.e.,

(42) $$p_{0}=exp\left(-\Upsilon \right)$$

□

Taking the derivative of $P_{n}$, we have

(43) $$P_{n}^{\prime }={\left(1-p_{0}\right)}^{\prime }=exp\left(-\Upsilon \right)\cdot \Upsilon^{\prime }$$

Recalling Formula (27), $\Upsilon^{\prime }$ can be written as

(44) $$\Upsilon^{\prime }=\lambda E{\left[T_{mac}\right]}^{\left(1\right)}$$

Since $E{\left[T_{mac}\right]}^{\left(1\right)}$ is bounded (proved by Appendix B, Lemma A1 ), we can assert that $\Upsilon^{\prime }$ is bounded. Consequently, we derive the following inequation

(45) $$P_{n}^{\prime }\leq exp\left(-\Upsilon \right)\cdot \Upsilon^{\prime }\leq 1\cdot \lambda \cdot A$$

where $E{\left[T_{mac}\right]}^{\left(1\right)}\leq A$ (*A* is a positive constant).

In other words, the first-order derivative of $P_{n}$ is bounded.

Further taking the derivative of $\tau_{u}\left(p\right)$, we have

(46) $${\left[\tau_{u}\left(p\right)\right]}^{\left(1\right)}=P_{n}^{\prime }\cdot \frac{E[R](p)}{E[X](p)}+P_{n}\cdot {\left[\frac{E[R](p)}{E[X](p)}\right]}^{\prime }$$

Noting that $\frac{E[R](p)}{E[X](p)}$ is a monotone, non-increasing function (proved by [23], Theorem 1) and ${\left[\frac{E[R](p)}{E[X](p)}\right]}^{\prime }\leq 0$ (demonstrated by Appendix C, Lemma A2), thus we can get the following inequation

(47) $$\begin{array}{c} {\left[\tau_{u}\left(p\right)\right]}^{\left(1\right)}\leq P_{n}^{\prime }\cdot \left[\frac{E[R](p)}{E[X](p)}|p=0\right]+P_{n}\cdot {\left[\frac{E[R](p)}{E[X](p)}|p=0\right]}^{\prime }\leq \lambda \cdot A\cdot \left[\frac{E[R](p)}{E[X](p)}|p=0\right] \end{array}$$

Reviewing Formula (17), $\Gamma^{\prime }\left(\tau_{u}\left(p\right)\right)$ can be written as

(48) $$\begin{array}{c} \Gamma^{\prime }\left(\tau_{u}\left(p\right)\right)=\left(N-1\right){\left(1-\tau_{u}\left(p\right)\right)}^{N-2}{\left[\tau_{u}\left(p\right)\right]}^{\left(1\right)}\leq \left(N-1\right)\lambda \cdot A\cdot \left[\frac{E[R](p)}{E[X](p)}|p=0\right]=C \end{array}$$

where *C* is a positive constant.

Hence, the first-order derivative of $\Gamma (\tau_{u}\left(p\right))$, i.e., $\Gamma^{\prime }\left(\tau_{u}\left(p\right)\right)$ is bounded.

**Theorem** **2.** *The sequence of $p_{\alpha }$ follows the relation of $\Gamma \left(\tau_{u}\left(p_{\alpha }\right)\right)\leq p_{\alpha }$.*

**Proof.**  We use inductive method to demonstrate this constrained condition. If $\Gamma \left(\tau_{u}\left(p_{\alpha }\right)\right)\leq p_{\alpha }$ holds, we can get the conclusion that $\Gamma \left(\tau_{u}\left(p_{\alpha +1}\right)\right)\leq p_{\alpha +1}$.

According to Theorem 1, we have proved that the first-order derivative of $\Gamma (\tau_{u}\left(p\right))$ is bounded, i.e., $\Gamma^{\prime }\left(\tau_{u}\left(p\right)\right)\leq C$, thus $\Gamma \left(\tau_{u}\left(p_{\alpha +1}\right)\right)-p_{\alpha +1}$ can be derived as

(49) $$\begin{array}{cc} \Gamma \left(\tau_{u}\left(p_{\alpha +1}\right)\right)-p_{\alpha +1}\; & \overset{a}{=}\Gamma \left(\tau_{u}\left(p_{\alpha +1}\right)\right)-\left[\left(1-\beta \right)\Gamma \left(\tau_{u}\left(p_{\alpha }\right)\right)+\beta p_{\alpha }\right]\\ \; & \overset{b}{=}\left[\Gamma \left(\tau_{u}\left(p_{\alpha +1}\right)\right)-\Gamma \left(\tau_{u}\left(p_{\alpha }\right)\right)\right]+\beta \left[\Gamma \left(\tau_{u}\left(p_{\alpha }\right)\right)-p_{\alpha }\right]\\ \; & \overset{c}{=}\Gamma^{\prime }\left(\tau_{u}\left(\varrho \right)\right)\left(p_{\alpha +1}-p_{\alpha }\right)+\beta \left[\Gamma \left(\tau_{u}\left(p_{\alpha }\right)\right)-p_{\alpha }\right]\\ \; & \overset{d}{\leq }C\left[p_{\alpha +1}-p_{\alpha }\right]+\beta \left[\Gamma \left(\tau_{u}\left(p_{\alpha }\right)\right)-p_{\alpha }\right]\\ \; & \overset{e}{=}C\left(1-\beta \right)\left[\Gamma \left(\tau_{u}\left(p_{\alpha }\right)\right)-p_{\alpha }\right]+\beta \left[\Gamma \left(\tau_{u}\left(p_{\alpha }\right)\right)-p_{\alpha }\right]\\ \; & \overset{f}{=}\left[\beta +C\left(1-\beta \right)\right]\left[\Gamma \left(\tau_{u}\left(p_{\alpha }\right)\right)-p_{\alpha }\right]\leq 0 \end{array}$$

where the “c”th line is derived based on Lagrange interpolation theorem, and ϱ is between $p_{\alpha }$ and $p_{\alpha +1}$.

Since $\Gamma (\tau_{u}\left(p\right))$ is continuous with respect to *p*, the bounded and non-increasing sequence must converge to a fixed point of *p*. In other words, we can guarantee that our proposed analytical model has the convergence property.□

## 5. Performance Evaluation

In this part, we developed simulations in Matlab. Table 1 and Table 4 provide the parameters, which would be used in simulation experiments. In the simulation, IEEE 1901 protocol is implemented by modifying the backoff counter window of 802.11 (executing backoff counter process), and adding a parallel deferral counter window (realizing the deferral counter process). In the experiment scene, a single-hop, star-shaped PLC network is constructed by *N* stations (adopting priority type CA0/CA1) and an access point (used to receive packets). The transmission distance from a station to the access point is Xm. The self-generating packets of each station employ a Poisson process, and the average rate is λ. Data packets are delivered from stations to the access point through a PLC channel that follows Rayleigh fading or Log-normal fading. The additive non-Gaussian noise feature is set to be Bernoulli-Gaussian distribution, and the data packet corresponds to a frame duration *D* ($D=\frac{L}{R}$). In addition, each simulation experiment contains 31 runs, where each run has 5000 packets for per station. The confidence interval of simulation results is [0.93Avg(.),1.07Avg(.)] (0.95 confidence), where Avg(.) represents the average simulation result. We choose Fixed-Point Iteration (FPI) method [41] to solve the proposed analytical model, and the terminate precision is set as $10^{-5}$ (due to the convergence property of the analytical model, using a reasonable numerical method is practicable).

Based on 1901’s specification, two PRS are used to declare the priority tone [28] before delivering the packet. After a RIFS, the station will receive an ACK frame if the transmission is successful; otherwise, a NACK frame. Between transmission attempt processes, there has a CIFS gap. If experiencing a collision, the station would wait for a duration of EIFS (corresponding to Formula (32) and Formulas (34) and (35)).

To measure the performance of IEEE 1901 protocol considering the impacts of ARQ scheme, channel fading type and additive non-Gaussian noise feature, we select four significant metrics: (1) System throughput *S*, (2) mean MAC service time $E[T_{mac}]$, (3) buffer blocking probability $P_{b}$, and (4) Successful transmission probability $P_{suc}$. The experiments totally have six groups: (1) the impact of network size, (2), the impact of packet arrival rate, (3) the impact of threshold SNR ζ, (4) the impact of the probability of impulsive noise component $P_{I}$, (5) the impact of the ratio of impulsive noise power K, and (6) the impact of transit buffer size *K*. Moreover, in following figures, RF (or LNF) denotes Rayleigh fading (or Log-normal fading), Ana (or Sim) represents analysis results (or simulation results).

### 5.1. The Impact of Network Size *N*

**Figure 5:**
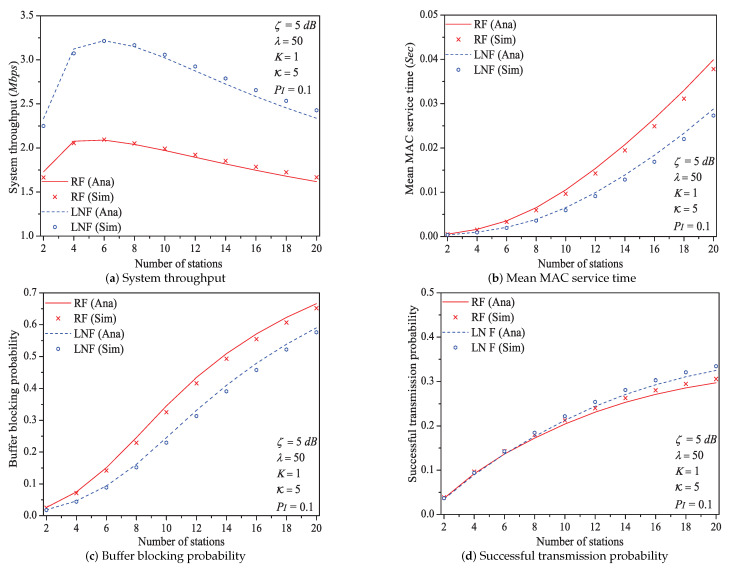
The MAC performance of 1901 protocol with different *N*.

In this simulation group, we set the threshold SNR ζ=5dB, probability of impulsive noise component $P_{I}=0.1$, ratio of impulsive noise power K=5, average packet arrival rate λ=50, transit buffer size K=1, and the number of stations *N* varies in [2,20]. Figure 5 shows the simulation and analysis results including the system throughput *S*, mean MAC service time $E[T_{mac}]$, buffer blocking probability $P_{b}$ and successful transmission probability $P_{suc}$ of the reliable PLC network with different *N*.

We can see that as the number of stations *N* increases, the system throughput increases first, then decreases (it is affected not only by the successful transmission probability but also by the expected slot time). With the increase of network size *N*. more stations would contend the medium. The data packet must wait a longer time duration to get the medium service, and the transit buffer of station is easier to be fully loaded. Consequently, the mean MAC service time and buffer blocking probability increase with the increasing network size *N*. As the network size has a positive influence on the performance of successful transmission probability $P_{suc}$, it increases with the increasing network size *N* (this conclusion can be demonstrated by Formula (30)).

Here is an example of simulation results (N∈[2,20]). the system throughput varies from 1.665Mbps to 2.095Mbps, then to 1.667Mbps under Rayleigh fading channel type; from 2.250Mbps to 3.213Mbps, then to 2.336Mbps under Log-normal fading channel type.

### 5.2. The Impact of Packet Arrival Rate λ

**Figure 6:**
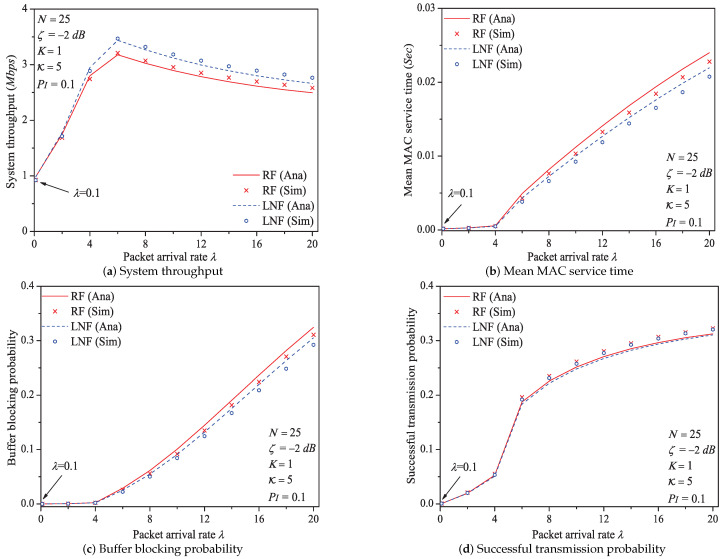
The MAC performance of 1901 protocol with different λ.

In this simulation group, we set the number of stations N=25, threshold SNR ζ=−2dB, probability of impulsive noise component $P_{I}=0.1$, ratio of impulsive noise power K=5, transit buffer size K=1, and the average packet arrival rate λ varies in [0.1,20]. Figure 6 shows the simulation and analysis results including the system throughput *S*, mean MAC service time $E[T_{mac}]$, buffer blocking probability $P_{b}$ and successful transmission probability $P_{suc}$ of the reliable PLC network with different λ.

We can see that as the packet arrival rate λ increases, the system throughput increases first, then slowly decreases. The mean MAC service time and buffer blocking probability increase with the increasing packet arrival rate. The reason is that as λ increases, the contention frequency of PLC medium is enhanced, and accordingly the collision probability increases. As a result, the data packet must wait a longer time duration to get the medium service, and the buffer of station is easier to be fully loaded. The successful transmission probability increases with the increasing λ. The most possible reason is that although the collision probability *p* and the transmission attempt probability $\tau_{u}\left(p\right)$ increase with the increasing λ; however, when λ varies in [0.1,20], the value of $\tau_{u}\left(p\right)$ is still smaller than $\frac{1}{N}$ (reviewing the analysis in Appendix A). Thus, the part $\tau_{u}\left(p\right)\left(1-p\right)$ in Formula (30) is still a monotone increasing function, which accordingly results the change rule of successful transmission probability.

Here is an example of analysis results (λ∈[0.1,20]). the system throughput varies from 0.983Mbps to 2.895Mbps, then to 2.495Mbps under Rayleigh fading channel type; from 0.979Mbps to 3.439Mbps, then to 2.664Mbps under Log-normal fading channel type.

### 5.3. The Impact of Threshold SNR ζ

**Figure 7:**
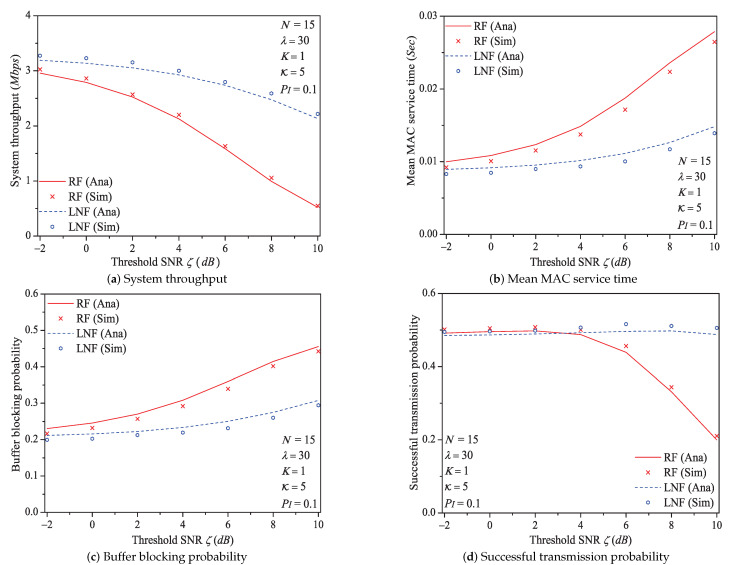
The MAC performance of 1901 protocol with different ζ.

In this simulation group, we set the number of stations N=15, probability of impulsive noise component $P_{I}=0.1$, ratio of impulsive noise power K=5, average packet arrival rate λ=30, transit buffer size K=1, and the required threshold SNR ζ varies in [−2dB,10dB]. Figure 7 shows the simulation and analysis results including the system throughput *S*, mean MAC service time $E[T_{mac}]$, buffer blocking probability $P_{b}$ and successful transmission probability $P_{suc}$ of the reliable PLC network with different ζ.

**Figure 8:**
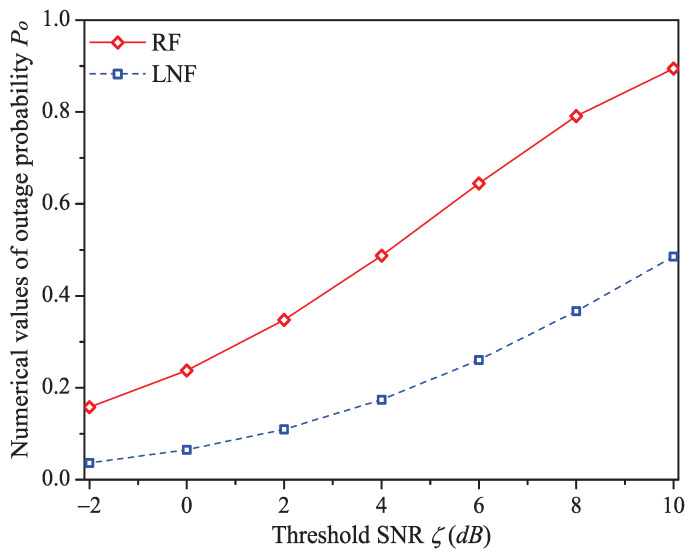
The numerical values of outage probability $P_{o}$ with different ζ.

We can see that as the threshold SNR ζ increases, the system throughput decreases. That is because as ζ increases, the packet has a higher probability to be received in outage (the change rule of numerical values of outage probability $P_{o}$ (Formula (9)) is shown in Figure 8), in other words, the attempt fragment is easier to be unsuccessful, and the transmission efficiency is accordingly decreased. The mean MAC service time and buffer blocking probability increase with the increasing ζ. The reason is that as ζ increases, the station occupying PLC channel may need more attempt fragments to accomplish a successful transmission (i.e., requiring more time). Hence, the data packet must wait longer in the station, and the buffer is easier to be fully loaded. The successful transmission probability in overall decreases (not monotone), since the increasing ζ can degrade the transmission efficiency.

Here is an example of simulation results (ζ∈[−2dB,10dB]). The mean MAC service time varies from 0.0092 s to 0.0265 s under Rayleigh fading channel type; from 0.0083 s to 0.0139 s under Log-normal channel fading type.

### 5.4. The Impact of the Probability of Impulsive Noise Component $P_{I}$

**Figure 9:**
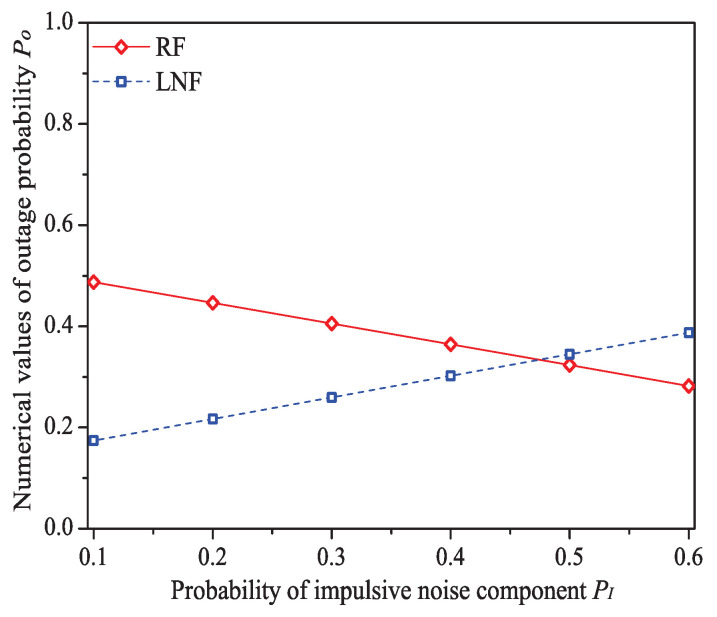
The numerical values of outage probability $P_{o}$ with different $P_{I}$.

In this simulation group, we set the number of stations N=15, threshold SNR ζ=4 dB, ratio of impulsive noise power K=5, packet arrival rate λ=30, transit buffer size K=1, and the probability of impulsive noise component $P_{I}$ varies in [0.1,0.6]. Figure 9 shows the simulation and analysis results including the system throughput *S*, mean MAC service time $E[T_{mac}]$, buffer blocking probability $P_{b}$ and successful transmission probability $P_{suc}$ of the reliable PLC networks with different $P_{I}$.

**Figure 10:**
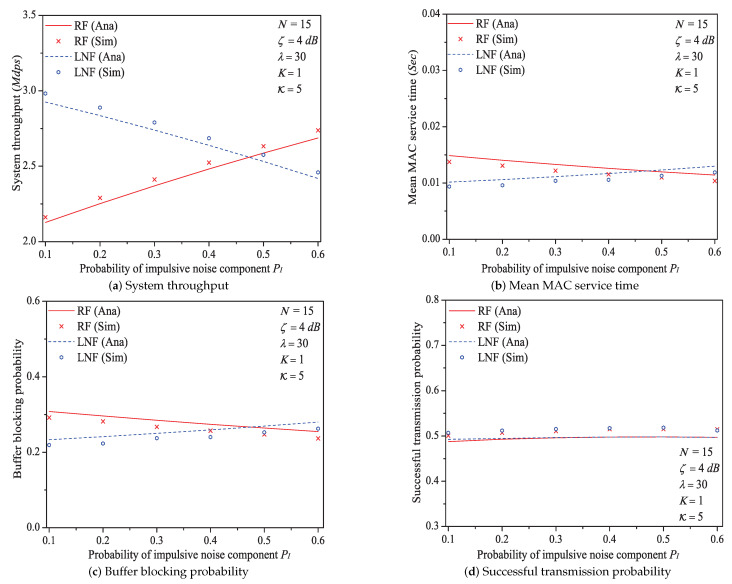
The MAC performance of 1901 protocol with different *P*_*I*_.

We can see that as the probability of impulsive noise component $P_{I}$ increases, the system throughput increases under Rayleigh fading channel type, but decreases under Log-normal fading channel type. The change rules of mean MAC service time and buffer blocking probability are opposite to that of system throughput. These conclusions illustrate that the outage probability $P_{o}$ (the change rule of numerical values of $P_{o}$ is shown in Figure 10) reflecting the state of PLC link layer can greatly affect the performance of 1901 MAC metrics. Taking the example of Rayleigh fading channel type, due to the decrease of $P_{o}$, the transmission efficiency increases, thus the system throughput increases with the increasing $P_{I}$. At the same time, packets in the station buffer only need shorter time to get the medium service and the transit buffer is hard to be fully loaded (hence the MAC service time and buffer blocking probability decrease under Rayleigh fading channel type). The value of successful transmission probability slightly increases at first, then slightly decreases (almost unchanged). The most possible reason is that although $P_{o}$ has a relatively large change, due to the introduction of ARQ scheme, the final change extent of operator $\left(1-P_{f}\right)$ in Formula (30) is minor. For example, the numerical value of $\left(1-P_{f}\right)$ under Rayleigh fading channel type merely varies from 0.944 to 0.994 for $P_{I}\in \left[0.1,0.6\right]$.

Here is an example of analysis results ($P_{I}\in \left[0.1,0.6\right]$). The mean MAC service time varies from 0.0149 s to 0.0114 s under Rayleigh fading channel type; from 0.0102 s to 0.0130 s under Log-normal channel fading type.

### 5.5. The Impact of the Ratio of Impulsive Noise Power K

**Figure 11:**
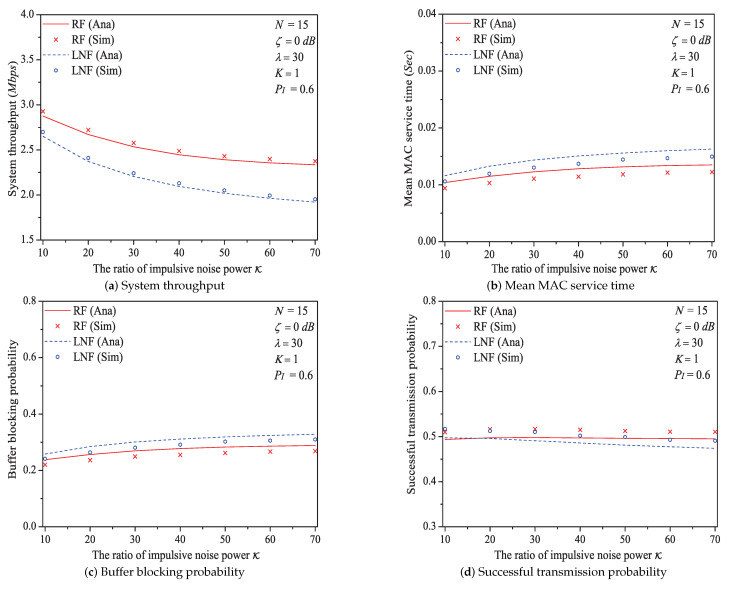
The MAC performance of 1901 protocol with different K.

In this simulation group, we set the number of stations N=15, threshold SNR ζ=0 dB, probability of impulsive noise component $P_{I}=0.6$, average packet arrival rate λ=30, transit buffer size K=1, and the ratio of impulsive noise power K varies in [10,70]. Figure 11 shows the simulation and analysis results including the system throughput *S*, mean MAC service time $E[T_{mac}]$, buffer blocking probability $P_{b}$ and successful transmission probability $P_{suc}$ of the reliable PLC network with different K.

**Figure 12:**
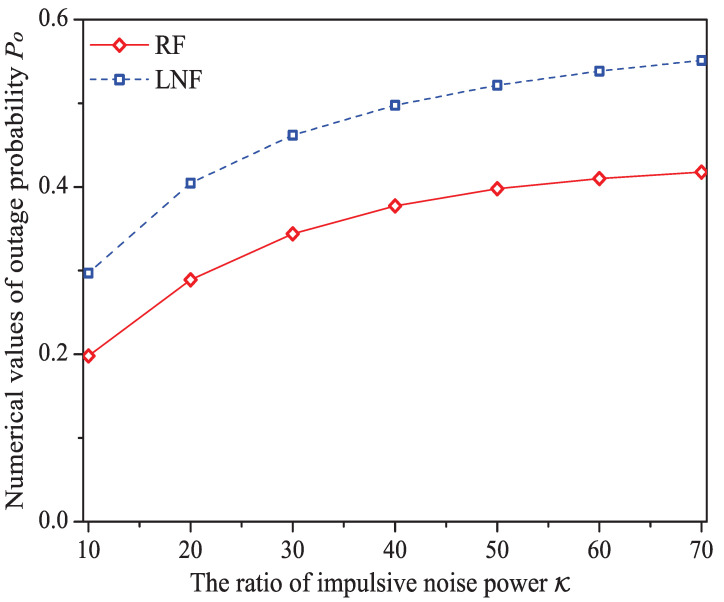
The numerical values of outage probability $P_{o}$ with different K.

We can see that as the ratio of impulsive noise power K increases, the system throughput decreases. The reason is that as K increases, the packet has a higher probability to be received in outage (the change rule of numerical values of outage probability $P_{o}$ is shown in Figure 12), in other words, the attempt fragment is easier to be unsuccessful, and the transmission efficiency is accordingly decreased. The mean MAC service time and buffer blocking probability increase with the increasing K. That is because as K increases, the station may need more attempt fragments to accomplish a successful transmission (i.e., requiring more time). Hence, the data packet must wait longer in the station, and the buffer is easier to be fully loaded. The successful transmission probability in overall decreases (not monotone), since the increasing K can degrade the transmission efficiency.

Here is an example of simulation results (K∈[10,70]). The buffer blocking probability varies from 0.220 to 0.268 under Rayleigh fading channel type; from 0.241 to 0.310 under Log-normal channel fading type.

### 5.6. The Impact of Transit Buffer Size *K*

**Figure 13:**
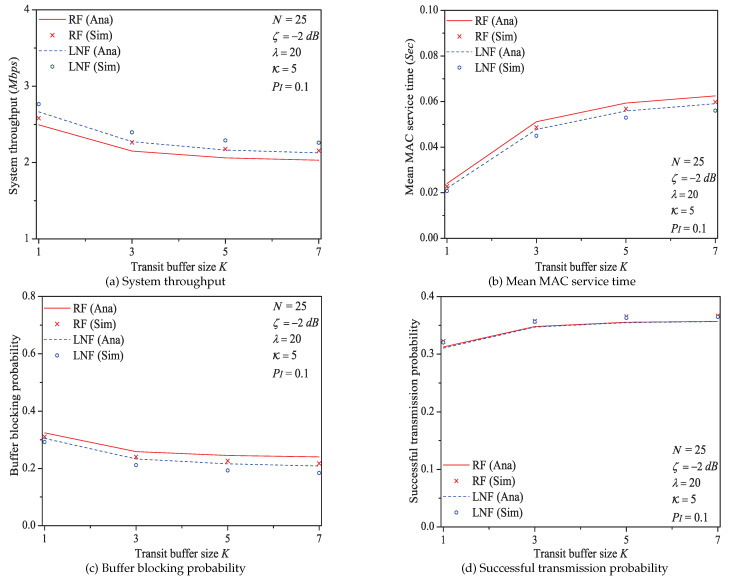
The MAC performance of 1901 protocol with different *K*.

In this simulation group, we set the number of stations N=25, threshold SNR ζ=−2 dB, probability of impulsive noise component $P_{I}=0.1$, average packet arrival rate λ=20, ratio of impulsive noise power K=5, and the transit buffer size *K* varies in [1,7] (the large value of *K* would greatly enhance the computation complexity, and the proposed analytical model needs to be solved by Lattice-Laplace method). Figure 13 shows the simulation and numerical analysis results including the system throughput *S*, mean MAC service time $E[T_{mac}]$, buffer blocking probability $P_{b}$ and successful transmission probability $P_{suc}$ of the reliable PLC network with different *K*.

We can see that the performance of system throughput, mean MAC service time, buffer blocking probability, and successful transmission probability is affected by the size of transit buffer *K*. It is easy to find that larger size of transit buffer would nor result superior performance of system throughput and MAC service time. However, with the increase of buffer size *K*, the transit buffer is no longer easy to be fully loaded. Consequently, the buffer blocking probability decreases with the increasing *K*, and the successful transmission probability is enhanced (since more packets can enter the transit buffer rather than directly being blocked).

Here is an example of analysis results (K∈[1,7]). The buffer blocking probability varies from 0.324 to 0.240 under Rayleigh fading channel type; from 0.305 to 0.209 under Log-normal channel fading type.

Through comparing the simulation and numerical analysis results shown in Figure 5, Figure 6, Figure 7, Figure 9, Figure 11 and Figure 13, we verify that our proposed analytical model can accurately measure the MAC performance of IEEE 1901 protocol for reliable PLC networks with ARQ scheme. Since the results for priority type CA2/CA3 contain the similar rules, we do not discuss them any more in this paper. In addition, the computational complexity of solving the theoretical model (using FPI method) is O(IT), where IT denotes the required iteration times (the detailed analysis is given in Appendix D)

## 6. Conclusions

In this paper, we put forward a MAC performance analysis model of IEEE 1901 protocol for reliable PLC networks with ARQ scheme. In the modeling process, we first construct a PLC channel model to reflect the impacts of detailed channel fading type (Rayleigh fading and Log-normal fading), non-Gaussian noise feature and ARQ scheme on data transmission at PLC’s link layer. Then, we use Renewal theory and Queueing dynamics to depict the transmission attempt behavior of executing IEEE 1901 protocol in unsaturated environment with finite transit buffer. On the base of combining these two models, we derive the closed-form expressions of typical 1901 MAC metrics for reliable PLC networks with ARQ scheme. Moreover, we prove that our proposed analytical model has the convergence property. To the best of our knowledge, this should be the first work to make the performance analysis of 1901 MAC protocol for reliable PLC networks considering link layer’s ARQ scheme. Through extensive simulations, we verify that the proposed analytical model can accurately evaluate the MAC performance of 1901 protocol for reliable PLC networks with ARQ scheme. Our work provides a significant foundation for optimizing the MAC performance of reliable PLC networks, and designing reliable PLC-based IoT.

Based on this work, we can start our future study in the following research directions: (1) Analyzing the MAC performance of 1901 for reliable PLC networks, which has hidden terminals and employs other network coding schemes; (2) Designing a MAC protocol for hybrid Power-line&Visible-light communication systems; (3) Optimizing the energy consumption for energy harvesting assisted reliable PLC networks with ARQ scheme.

## Figures and Tables

**Table 1:** The maximum backoff counter $W_{k}$ [16] and the value of $d_{k}$ [16] for 1901 protocol.

PriorityClass:	CA0	CA1	CA2	CA3
backoff stage *k*	$W_{k}$	$d_{k}$	$W_{k}$	$d_{k}$
1	8	0	8	0
2	16	1	16	1
3	32	3	16	3
4	64	15	32	15

**Table 2:** Summary of the works for IEEE 1901 protocol analysis (including our current work).

Study	Mathematics Tool	Network Topology	Traffic Type	Considered Factors in the Modeling Process
Jung [21]	Semi-Markov chain	Single-hop	Homogeneous	Infinite buffer size; Infinite traffic load
Vlachou [22]-[23]	Renewal theory	Single-hop	Homogeneous	Infinite buffer size; Infinite traffic load
Vlachou [24]-[25]	Renewal theory	Single-hop	Homogeneous	Infinite buffer size Infinite traffic load; Performance optimization
Vlachou [26]	Renewal theory & Control theory	Single-hop	Homogeneous	infinite buffer size; infinite traffic load; dynamics stability analysis
Cano [27]	Renewal process	Single-hop	Homogeneous	Infinite buffer size; Infinite traffic load
Hao [28]	Discrete Markov chain & Queueing theory	Single-hop	Homogeneous & Heterogeneous	Finite buffer size; Finite traffic load
Hao [29]	Discrete Markov chain & Jackson Queueing system	Multi-hop	Homogeneous	Finite buffer size; Finite traffic load
Our current work	Renewal theory & Queueing dynamics (Z transforms)	Single-hop	Homogeneous	Finite buffer size; Finite traffic load; **Noise feature**; **Solvable proof**; **Coding scheme of link layer**

**Table 3:** Notation list relevant to essential results derived by the analytical model.

Notation	Definition
*N*	Number of stations
*M*	Number of attempt fragments
*X*	Transmission distance
*K*	Transit buffer size
$h_{R}/h_{L}$	Channel gain of Rayleigh/Log-normal distribution
$P_{tr}$	Transmission power
$\sigma_{g}^{2}/\sigma_{b}^{2}/P_{I}/K$	Parameters of the PLC noise (following Bernoulli-Gaussian distribution)
$\sigma_{R}^{2}$	Scale parameter of Rayleigh distribution
$\sigma_{L}^{2}/\mu_{L}$	Scale parameters of Log-normal distribution
ξ	Signal-to-Noise ratio SNR
ζ	Required minimum SNR (threshold SNR)
$f_{\xi }^{R}\left(.\right)/f_{\xi }^{L}\left(.\right)$	Probability density function of SNR using Rayleigh/Log-normal fading channel
$F_{\xi }^{R}\left(.\right)/F_{\xi }^{L}\left(.\right)$	Cumulative distribution function of SNR using Rayleigh/Log-normal fading channel
$P_{o}$	Probability that the transmission of PLC link fails
$P_{s}\left(x\right)$	Probability that one transmission attempt process of PLC link successes and needs *x* attempt fragments
$P_{f}$	Probability that one transmission attempt process of PLC link fails
$\tau_{s}\left(.\right)/\tau_{u}\left(.\right)$	Transmission attempt probability in saturated/unsaturated environment
E[R](.)	Expected number of attempts by executing one complete procedure of 1901 protocol
E[X](.)	Expected number of time slots spent in executing one complete procedure of 1901
$b_{i}$	Expected number of time slots spent by a station at backoff stage *i*
$\chi_{i}$	Probability that a station at backoff stage *i* must jump to the next stage
ρ/p	Conditional collision probability in saturated/unsaturated environment
$X_{h}^{i}$	Probability of triggering DCP of 1901 and spending no more than *h* time slots at stage *i*
$P_{n}$	Steady state probability that station buffer is none-empty
$p_{tr}$	Probability that at least one station tries to a transmission in the considered slot time
$p_{idle}$	Probability that PLC channel is idle
Υ	Traffic intensity
$p_{a}$	Probability of *a* packets in the transit buffer
$E[T_{mac}]$	Mean MAC service time
$p_{suc}\left(x\right)$	Probability that a station successfully transmits its HOL packet and uses *x* attempt fragments
$P_{suc}$	Successful transmission probability
$p_{drop}$	Probability that a packet is finally dropped in one transmission attempt process
$T_{s}\left(x\right)$	Time duration that a successful transmission using *x* attempt fragments
$T_{c}$	Time duration that channel is busy due to the collision
$T_{drop}$	Time duration of “dropping packet”
$E_{slot}$	Expected slot time
*S*	System throughput
$P_{b}$	Buffer blocking probability

**Table 4:** System parameters of reliable PLC networks.

MAC Layer Parameters	Size
Medium bit rate (*R*)	10 Mbps [28,29]
Slot (σ)	35.84 us [16]
Priorityslot(PRS)	35.84 us [16]
CIFS	100.00 us [16]
RIFS	140.00 us [16]
NACK	110.48 us [16]
ACK	110.48 us [16]
EIFS	2920.64 us [16]
*L* (packet size)	5000.00 bits [20,28]
*m* (maximum backoff stage)	4 [16]
Priority type	CA0/CA1 [16]
**Link layer parameters (normalized)**	**Size**
*M* (number of attempt fragments)	4 [13,14]
*X* (transmission distance)	8 m [13,14]
$\Omega_{1}$	1 [30,31]
$\Omega_{2}$	0.5 [30,31]
α (loss exponent)	2.6 [30,31]
$\sigma_{g}^{2}$	0.1 [30,31]
$\sigma_{R}^{2}$	1 [30,31]
$\sigma_{L}^{2}$	0.5 [30,31]
$\mu_{L}$	0.1 [30,31]

**Figure A1:**
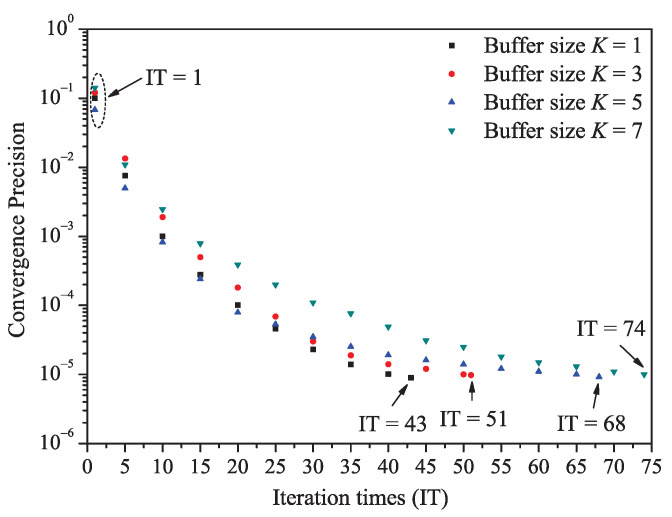
The relationship between iteration times and convergence precision under the impact of transit buffer size *K*.

## Data Availability

Data sharing is not applicable to this article.

## Appendix A. The Theoretically Optimal Value of Successful Transmission Probability P suc

Let the maximum attempt fragment be $M_{max}$. Reviewing Formula (30), it can be extended as a binary function $F(M,\tau_{u})$ ($\tau_{u}$ is the simplified format of $\tau_{u}\left(p\right)$), i.e.,

$$F\left(M,\tau_{u}\right)=N\tau_{u}{\left(1-\tau_{u}\right)}^{N-1}\left(1-P_{o}^{M}\right)$$

Thus, we take the partial derivative for $F(M,\tau_{u})$

(A1) $$\begin{array}{c} \frac{\partial F_{R}\left(M,\tau_{u}\right)}{\partial M}=-\ln\left(P_{o}\right)P_{o}^{M}\cdot N\tau_{u}{\left(1-\tau_{u}\right)}^{N-1};\;M\in \left[1,M_{max}\right] \end{array}$$

(A2) $$\begin{array}{cc} \; & \frac{\partial F_{R}\left(M,\tau_{u}\right)}{\partial \tau_{u}}=N\left(1-P_{o}^{M}\right)\left(1-N\tau_{u}\right){\left(1-\tau_{u}\right)}^{N-2} \end{array}$$

Clearly, $\frac{\partial F_{R}\left(M,\tau_{u}\right)}{\partial M}>0$ ($0<P_{o}<1$), $\frac{\partial F_{R}\left(M,\tau_{u}\right)}{\partial \tau_{u}}$ has an extreme value point, i.e., $\tau_{u}=\frac{1}{N}$, therefore, the theoretically optimal value of successful transmission probability can be written as

(A3) $$P_{suc-optimal}=\left(1-P_{o}^{M_{max}}\right){\left(1-\frac{1}{N}\right)}^{N-1}$$

For N→+∞, $P_{suc-optimal}$ can be further expressed as

(A4) $$\lim_{N\to +\infty }P_{suc-optimal}=\left(1-P_{o}^{M_{max}}\right)exp\left(-1\right)$$

## Appendix B

**Lemma** **A1.**
*$E{\left[T_{mac}\right]}^{\left(1\right)}$ Is Bounded*

**Proof.** First, we rewrite $E[T_{mac}]$, and it can be expressed as

(A5) $$E\left[T_{mac}\right]=\sum_{j=0}^{\infty }Prb\left\{T_{mac}=jE\left[slot\right]\right\}\left[jE\left[slot\right]\right]$$

Thus, we have

(A6) $$\begin{array}{c} E{\left[T_{mac}\right]}^{\left(1\right)}=\sum_{j=0}^{\infty }\left\{j{\left[Prb\left\{T_{mac}=jE\left[slot\right]\right\}\right]}^{\prime }\left[E\left[slot\right]\right]+\left[jPrb\left\{T_{mac}=jE\left[slot\right]\right\}\right]{\left[E\left[slot\right]\right]}^{\prime }\right\} \end{array}$$

It is easy to find $\left[jPrb\left\{T_{mac}=jE\left[slot\right]\right\}\right]$ and ${\left[jPrb\left\{T_{mac}=jE\left[slot\right]\right\}\right]}^{\prime }$ are both monotone, increasing functions (derived by Formula (23)), thus, we only need to judge whether E[slot] and ${\left[E\left[slot\right]\right]}^{\prime }$ are bounded.

Recalling Formula (37), we can get the following inequation

(A7) $$\begin{array}{c} E\left[slot\right]\leq \sigma +\sum_{x=1}^{M}\left[1-{\left(1-\frac{1}{N}\right)}^{N-1}\right]P_{s}\left(x\right)T_{s}\left(x\right)+T_{dp}\left[1-{\left(1-\frac{1}{N}\right)}^{N-1}\right]P_{f}+T_{c} \end{array}$$

Taking the derivative of E[slot], we have

(A8) $$\begin{array}{c} {\left[E\left[slot\right]\right]}^{\prime }=\left(T_{c}-\sigma \right)\frac{N}{N-1}{\left(1-p\right)}^{\frac{1}{N-1}}+\left(\sum_{x=1}^{M}T_{s}\left(x\right)P_{s}\left(x\right)+T_{dp}P_{f}-T_{c}\right)\left[N\left(\frac{N}{N-1}{\left(1-p\right)}^{\frac{1}{N-1}}-1\right)\right] \end{array}$$

Since $\frac{N}{N-1}\leq 2$, we can derive the following inequation for ${\left[E\left[slot\right]\right]}^{\prime }$

(A9) $${\left[E\left[slot\right]\right]}^{\prime }\leq 2\left(T_{c}-\sigma \right)+N\left[\sum_{x=1}^{M}T_{s}\left(x\right)P_{s}\left(x\right)+T_{dp}P_{f}-T_{c}\right]$$

From the above analysis, we demonstrate that both E[slot] and ${\left[E\left[slot\right]\right]}^{\prime }$ are bounded. Accordingly, Formula (A6) can be extended as (*A* denotes a positive constant)

(A10) $$\begin{array}{cc} \; & E{\left[T_{mac}\right]}^{\left(1\right)}\leq \sum_{j=0}^{\infty }j{\left|\left[Prb\left\{T_{mac}=jE\left[slot\right]|p=1\right\}\right]\right|}^{\prime }\cdot \\ & |\sigma +\sum_{x=1}^{M}\left[1-{\left(1-\frac{1}{N}\right)}^{N-1}\right]P_{s}\left(x\right)T_{s}\left(x\right)+T_{dp}\left[1-{\left(1-\frac{1}{N}\right)}^{N-1}\right]P_{f}+T_{c}|+\\ \; & \sum_{j=0}^{\infty }j|\left[Prb\left\{T_{mac}=jE\left[slot\right]|p=1\right\}\right]|\cdot [2\left(T_{c}-\sigma \right)+N|\sum_{x=1}^{M}T_{s}\left(x\right)P_{s}\left(x\right)+T_{dp}P_{f}-T_{c}\left|\right]=A \end{array}$$

Namely $E{\left[T_{mac}\right]}^{\left(1\right)}$ is bounded.□

## Appendix C

**Lemma** **A2.**
${\left[\frac{E[R](p)}{E[X](p)}\right]}^{\prime }\leq 0$

**Proof.** Directly using the conclusions proved in [23] (THEOREM 1), ${\left[\frac{E[R](p)}{E[X](p)}\right]}^{\prime }$ can be expressed as

(A11) $${\left[\frac{E[R](p)}{E[X](p)}\right]}^{\prime }=-\frac{Q^{\prime }\left(p\right)}{Q^{2}\left(p\right)}>0$$

where Q(p) is denoted as

(A12) $$Q\left(p\right)=\frac{E[X](p)}{E[R](p)}={\left[\tau_{u}\left(p\right)\right]}^{-1}$$

$Q^{\prime }\left(p\right)$ has an inequality relation derived by Formula (8) and Formula (9) of [23], i.e.,

(A13) $$\begin{array}{cc} \; & Q^{\prime }\left(p\right)\geq -\sum_{i=1}^{m-2}\left(1-\chi_{i}^{\prime }\right)\frac{1}{\chi_{i}}[\sum_{j=i+1}^{m-1}\left(\left(B_{j}+1\right)\left(1-\chi_{i}\right)\prod_{t=0}^{j-1}\chi_{t}\right)\\ \; & -\left(B_{i}+1\right)\prod_{j=1}^{i}\chi_{j}+\prod_{j=0}^{m-1}\chi_{j}\left(B_{m}+1\right)]\geq 0 \end{array}$$

where $B_{i}$ is expressed as

(A14) $$B_{i}=\frac{b_{i}}{\sum_{r=1}^{d_{i}}\frac{1}{W_{i}}+\sum_{r=d_{i}+1}^{W_{i}}\left(1-X_{r}^{i}\right)}-1$$

Since Q(p) is a monotone, non-decreasing function [23] (THEOREM 1) and $Q^{\prime }\left(p\right)\geq 0$, we have

(A15) $${\left[\frac{E[R](p)}{E[X](p)}\right]}^{\prime }=-\frac{Q^{\prime }\left(p\right)}{Q^{2}\left(p\right)}\leq -Q^{\prime }\left(p\right)\cdot \left[\frac{1}{Q^{2}\left(p\right)}|p=0\right]\leq 0$$

In other words, ${\left[\frac{E[R](p)}{E[X](p)}\right]}^{\prime }$ is bounded. □

## Appendix D. Computation Complexity of Solving the Theoretical Model with FPI Method

To solve the numerical results of the MAC metrics, we need to calculate the correct value of *p*. In our paper, Fixed-Point Iteration (FPI) method is employed, and the calculation process is given as follows (Table A1):

**Table A1:** Fixed-point iteration method.

	1: Select any value of p(0) (0 denotes the iteration times), which are not absurd (i.e., p(0)∈(0,1))
	2: Calculate the values of E[slot](0), $E\left[T_{mac}\right]\left(0\right)$ and $P_{n}\left(0\right)$ in order (based on our theoretical model)
	3: Find out the value of τ(p)(0) through using Formula (14) based on step 2’s conclusion
	4: Derive the new p(1) by substituting τ(p)(0) into Formula (17)
	5: Repeat the above process (through iterating calculation)
	6: If |p(φ+1)−p(φ)|<θ (θ is the terminate precision we defined), replace *p* with p(φ+1), and then calculate the numerical values of other MAC metrics.

Clearly, if we set a reasonable θ, we can finally derive the results of the theoretical model in a finite number of iteration times IT (a constant). Since we have assumed that the transmission attempt process of each station follows the decoupling assumption [23], the computational complexity of solving our theoretical model can be written as

(A16) O(IT)

It should be noted that the number of iteration times is affected by the system parameters setting. Here we give an example. Figure A1 shows the relationship between iteration times of solving the proposed theoretical model and the convergence precision (equal to |p(i+1)−p(i)|;i∈[0,φ]) for the last experiment group (“impact of transit buffer size *K*”). We can find that the iteration times and the buffer size have a positive relationship, in other words, the large value of buffer size *K* would enhance the iteration times (decreasing the convergence speed). The reason is that the large buffer would enhance the random matrix’s dimension given by Formulas (18) and (19).
